# Data-Driven Technologies as Enablers for Value Creation in the Prevention of Surgical Site Infections: a Systematic Review

**DOI:** 10.1007/s41666-023-00129-2

**Published:** 2023-02-27

**Authors:** Luís Irgang, Henrik Barth, Magnus Holmén

**Affiliations:** School of Business, Innovation and Sustainability - Department of Engineering and Innovation, Halmstad University, Halmstad, Sweden

**Keywords:** Healthcare technology, Surgical site infections, Infection prevention and control, Value-based care, Technology implementation, Systematic review

## Abstract

Despite the advances in modern medicine, the use of data-driven technologies (DDTs) to prevent surgical site infections (SSIs) remains a major challenge. Scholars recognise that data management is the next frontier in infection prevention, but many aspects related to the benefits and advantages of using DDTs to mitigate SSI risk factors remain unclear and underexplored in the literature. This study explores how DDTs enable value creation in the prevention of SSIs. This study follows a systematic literature review approach and the PRISMA statement to analyse peer-reviewed articles from seven databases. Fifty-nine articles were included in the review and were analysed through a descriptive and a thematic analysis. The findings suggest a growing interest in DDTs in SSI prevention in the last 5 years, and that machine learning and smartphone applications are widely used in SSI prevention. DDTs are mainly applied to prevent SSIs in clean and clean-contaminated surgeries and often used to manage patient-related data in the postoperative stage. DDTs enable the creation of nine categories of value that are classified in four dimensions: cost/sacrifice, functional/instrumental, experiential/hedonic, and symbolic/expressive. This study offers a unique and systematic overview of the value creation aspects enabled by DDT applications in SSI prevention and suggests that additional research is needed in four areas: value co-creation and product-service systems, DDTs in contaminated and dirty surgeries, data legitimation and explainability, and data-driven interventions.

**Supplementary Information:**

The online version contains supplementary material available at 10.1007/s41666-023-00129-2.

## Background

The collection and use of data have become increasingly powerful because of the rapid progress of information and communication technologies, including sensors, computers, software, and mobile gadgets. In the healthcare industry, the management of patient-related data contributes to the delivery of more reliable healthcare services and allows hospitals to better distribute resources, organise healthcare routines, and predict adverse events [1]. However, the use of data is often suboptimal, partly due to the complexities associated with the collection and use of healthcare data [2] and partly because of the need for organisational adaptation to exploit the potential of data-driven technologies to provide high-quality care [3, 4].

Data-driven technologies—DDTs (so-called digital technologies of Industry 4.0)—enable digital, networked, automated, and intelligent processes such as digital platforms, smart sensors, and artificial intelligence systems [5, 6]. DDTs are applied in various data management functions, including the generation and capture of data, data transmission and sharing, data conditioning, storage and processing, data application, and data security and protection [7]. With the advent of Internet of Things (IoT) and Big Data, DDTs have gained momentum, as they allow the management of large heterogeneous datasets, which may contribute to the formulation of problems in different contexts [7, 8]. Scholars claim that DDTs open a new data-centric paradigm where data is the primary output that can foster new innovations and trigger dramatic changes in organisations [5, 9].

Research on the use of DDTs in healthcare have gained prominence in the last decades, especially on the area of prevention of surgical site infections (SSIs). A recent literature review indicates that DDTs can improve surgical precision, reduce manpower, support decision-making, and increase surgical safety [10]. Studies show that DDTs can provide insights on large and complex real-time dataset on ongoing operations, which would be valuable feedback for patients, doctors, nurses, and hospital managers on how to identify and reduce SSI risks [11, 12]. Successful real-time data analysis of SSIs provides new potential treatments and procedures and allows for new organisational responses based on direct feedback loops [13].

Despite the contributions from recent studies, the literature remains fragmented, and research findings are often context specific or ambiguous [14]. Prior literature is either patient-centric, focusing on clinical aspects [15], or driven by a technological-centric approach, focusing on technical aspects such as the implementation and operation of technologies [16, 17]. However, studies show that creating value from DDTs goes beyond considering individual and technical factors but relies on the integration of complementary resources and capabilities [5, 18]. Currently, an understanding on how DDTs enhance value creation in transformative knowledge-intensive service industries (e.g. healthcare) and how data is used to maximise service quality remains a challenge, and additional research is needed to investigate how multiple actors in value chains coordinate activities and resources to benefit from DDTs [3]. From a research perspective, we need to understand the value creation potential of DDTs in hospital settings [3] and specifically how DDTs can be a driver for new organisational responses to reduce SSI incidence. Unfortunately, there is scarce information on how to create value from DDT applications to prevent SSIs, and systematic studies about DDTs and their potential in hospital settings and how to fully grasp the benefits of using data to reduce the burden of SSIs are still absent [19, 20].

Against this backdrop, the objective of this study is to explore how DDTs enable value creation in the prevention of SSI. We adopted a systematic literature review approach [21] to identify the DDTs used in the prevention of SSIs, describe the context where DDTs are applied in SSI prevention, characterise the how data is managed in SSI prevention by DDTs, and analyse the type of value that is created, which actors benefit from the value created and how they benefit from it.

## Theoretical Concepts

### Surgical Site Infections (SSIs)

SSIs, also known as surgical wound infections or postoperative infections, are a major cause of prolonged hospitalisation and increased morbidity and mortality [22]. They occur up to 30 days after a surgical intervention (or within 1 year in patients with prosthetic implant) and are the most frequent of all hospital infection cases, affecting around 800,000 patients every year in Europe [23]. SSIs are among the costliest healthcare events, where one single case can generate additional costs of up to 49,000 dollars [24], leading to heavy financial losses.

SSI prevention relies on the mitigation of multiple patient-related (endogenous) and procedure-related (exogenous) risk factors. Endogenous risk factors include older age, pre-existing colonisation or infection, diabetes, and malnutrition, while external risk factors include the duration of the operation, inadequate sterilisation of surgical instruments, inappropriate behaviour of the surgical team, and contaminated operating room environment [22, 25].

SSI occurrence is difficult to predict, as it depends mostly on the relative probability of a surgical wound to get infected. Surgeries are classified in four categories according to the likelihood and degree of wound contamination at the time of operation: (i) *clean*, when no inflammation is encountered and the respiratory, alimentary, genital, or urinary tracts are not entered (e.g. eye surgery, neurosurgery, vascular surgery); (ii) *clean-contaminated*, when the respiratory, alimentary, genital, or urinary tracts are entered under controlled conditions and without unusual contamination (e.g. caesarean section, dental surgery); (iii) *contaminated*, when it includes open or fresh accidental wounds, or when it involves repairing or removing an internal organ, with risk for the blood and other fluids to spill into the wound (rectal surgery, appendicectomy); and (iv) *dirty*, when wounds have infectious pathogens already present at the time of the surgery (e.g. preoperative perforation of respiratory tract) [26, 27].

Despite the advances in modern medicine, preventing SSIs remains a major challenge for frontline workers and healthcare managers. Prior studies advocate that the strategies to prevent SSIs should be focused on minimising both endogenous and exogenous risks factors over three stages of patient care, i.e. preoperative, intraoperative, and postoperative [28]. Preoperative strategies are tailored to improve the patient’s defences against infections with the aim to ensure that the operating room is not contaminated, and the surgical team is committed to hygiene measures and the use of personal protective equipment. Intraoperative strategies are related to the maintenance of adequate environmental conditions in the operating room during the surgical procedures and the appropriate behaviour of the surgical team and use of correct surgical practices. Postoperative strategies embrace the hygiene practices and antibiotic treatment aimed to wound healing and patient recovering, including the periods right after the surgery, post discharge, and potential readmission [29]. Studies claim that surveillance must be classified as the fourth stage in SSI prevention, due to its potential to provide data that can be used to contrast SSI rates and compare surgical outcomes among surgeons or facilities, which ultimately contributes to identify failures and opportunities for improvement in the prevention measures [22].

### Value Creation in Healthcare

The concept of value has been widely used in healthcare industry to drive the development and implementation of new products, technologies, services, and practices to improve the quality of patient care [30, 31]. In the literature, value is a multifaceted concept with little in common across different research streams. Much of the discussion is grounded on the idea that value refers to the health outcomes achieved per dollar spent [32], thus representing the measurement of efficiency of healthcare delivered. This approach frames value creation around the patients, which in turn determines how other stakeholders in the value chain should be rewarded [33]. In sum, providers create value by adding features to products and services that increase the benefits for patients or by reducing the efforts that patients need to undertake to purchase or use a good or a service [34].

In contrast, service marketing and innovation management scholars advocate that value is subjectively perceived by the customers or users rather than objectively measured in monetary terms [35]. This view is anchored on the idea that value resides in individual’s experiences with the consumption of products and services, and that is moderated by the individual’s expectations and by the social context [36, 37]. This approach emphasises that value is created from social interactions and resource integration of multiple actors to obtain mutual benefits [38]. In healthcare, the dynamics and complexity of the patient care cycle involve the participation of multiple actors with often divergent interests, from medical and nursing staff, insurance companies, patients, and patients’ families [39]. This implies a broad understanding that, although value chains in healthcare are designed to meet patients’ needs [40], other actors also seek to obtain benefits, so value must be approached from a multi-actor perspective.

Along with a multi-actor understanding of value, innovation management literature focuses on how to create value from technological applications in healthcare and who can benefit from it [3, 41]. In this regard, technologies are viewed as a solution to contribute to patient care routines, to improve organisational processes and stakeholder relationships, and to create new business opportunities [42]. Here, value creation in healthcare entails a stream of activities carried out by individuals or organisations motivated by the integration of knowledge, skills, physical structure, and financial resources to facilitate the generation of tangible and intangible benefits [43].

To further contribute to the conceptualisation and characterisation of value and value creation, scholars developed multiple approaches to assess how value is perceived by individuals or organisations. A well-accepted approach classifies value created in four dimensions: cost/sacrifice, functional/instrumental, experiential/hedonic, and symbolic/expressive [44]. *Cost/sacrifice* value is related to when customers/end users’ perceived benefits exceed the efforts needed to buy, own, and use a product or service [40]. It involves monetary costs (e.g. price, distribution costs, costs of operation, use, and maintenance), non-monetary costs (e.g. psychological and cognitive costs), and risks (e.g. personal, operational, financial, or strategic risks) [44]. *Functional/instrumental* value is related to the accuracy of attributes and features of a product or service, its appropriate performance, and its satisfactory outcomes [44]. This type of value is often associated to the extent which a technology can solve a relevant problem (usefulness) and generate potential benefits [45]. *Experiential/hedonic* value refers to the sensory, emotional, epistemic, social, and relational values [44]. This type of value can be related to positive feelings, sensations, and emotions that a customer or end-user experience when buying or using a product or service [46]. *Symbolic/expressive* value embraces the advantage of strengthened social contacts, knowledge sharing, and innovation [44]. This value is concerned to the social and conditional meaning, the personal meaning, the self-identity/worth, and the self-expression of groups and individuals [47].

## Methods

To identify relevant literature on value creation from DDT applications in SSI prevention from a multi-actor approach, we conducted a systematic literature review study, which allows to identify, evaluate, and synthesise research results that can contribute to determine gaps within the extant research and provide guidance for further research activities [21]. Similar to Hernandez et al. [13], we followed the Preferred Reporting Items for Systematic Reviews and Meta-analyses Protocol (PRISMA) statement [48] to document the review process through a detailed systematic review protocol and a flow diagram.

### Search Strategy and Identification

We conducted a comprehensive search on scientific journals indexed in bibliographic databases. Given the multidisciplinary nature of the study objective, journals indexed in the Scopus, Web of Science, MEDLINE, ProQuest, PubMed, ABI/Inform Global, and Cochrane electronic databases were included. The databases cover peer-reviewed scientific studies in the fields of innovation management, information systems, health, and medical sciences.

We included in the search a list of terms and synonyms related to SSI and DDTs based on taxonomies and classifications attributed to surgical site infections (e.g. surgical wound infection, postoperative infection) [22] and DDTs (e.g. information and communication technology, digital technology) [7, 8, 49]. Search mechanisms such as truncations, proximity parameters (e.g. Medical Subject Headings and ProQuest thesaurus) and Boolean operators were combined to expand the search scope. The list was refined based on several rounds of pilot tests through scoping searches, where the search terms were extensively tested, labelled, and re-labelled to ensure a fulsome search for the review. The final list of search terms was then defined based on several discussion rounds between the authors (see Online Resource 1).

We conditioned the presence of the keywords in the articles’ abstract, title, or keywords to refine the search. We searched for peer-reviewed articles written in English and published in journals. In line with similar review articles [50], we set a preliminary time frame of 10 years (2011–2020) as the baseline for our search to gain robust insights into relevant empirical studies on DDTs and SSI prevention. Nevertheless, given the recent increased interest in DDT applications in healthcare, we selected 2021 as the cut-off year to have an updated and comprehensive picture of the recent literature. Therefore, articles published from 1 January to 20 November 2021 were also included for inclusion.

We developed a search string tailored to Scopus database, which was adapted according to the parameters of the other six databases (see Online Resource 2). The search was performed between August and November 2021. Altogether, the strings resulted in an initial set of 2597 articles. These articles were exported to EndNote X9 software, which allowed the identification and exclusion of 1104 duplicated articles. Later, the 1493 remaining unique articles were exported to Rayyan, which is a free online tool used to assist the initial screening of abstracts and titles.

### Screening and Eligibility Criteria

The initial screening took place in October 2021, and it was performed in a blind mode by the first and second author of the study. To avoid bias, the authors independently screened the titles and abstracts of all 1493 articles and checked their eligibility for the study based on inclusion and exclusion criteria. Inclusion criteria were as follows: (i) empirical studies addressing the use of DDTs tailored primarily to the prevention of SSIs. Exclusion criteria were as follows: (i) conference proceedings, books, book chapters, and dissertations; (ii) non-empirical studies, guidelines, and protocols; (iii) studies focusing on other postoperative complications and infections than SSIs; and (iv) studies including other technologies and solutions than DDTs.

The first screening resulted in the exclusion of 1332 articles. The first two authors classified 101 articles as potentially eligible for the review, while they disagreed in the classification of 60 articles. The third author, a senior researcher, reviewed the titles and abstracts of the 60 articles and classified 52 as potentially eligible for the study. Finally, a total of 153 articles were considered potentially eligible for the study.

### Study Selection

**Fig. 1:**
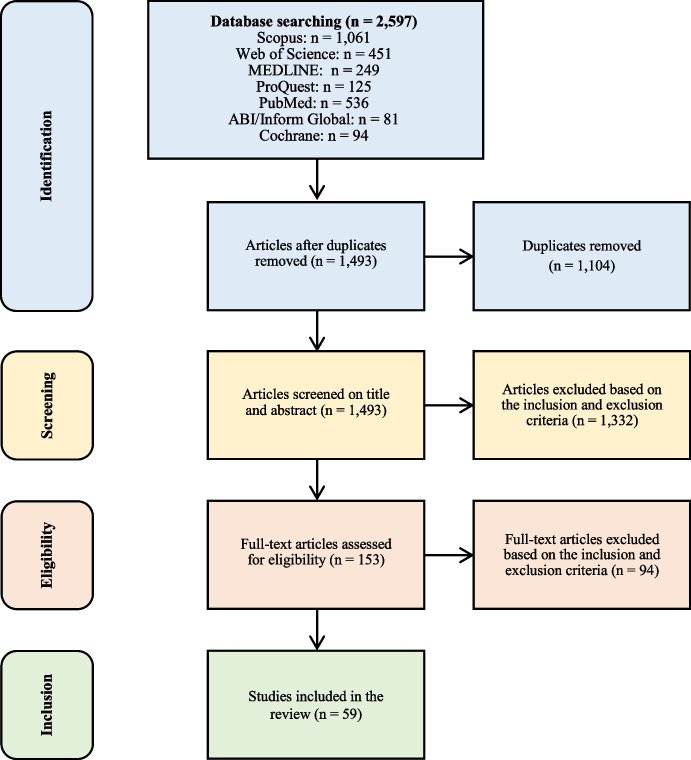
PRISMA flowchart of the selection process

The full texts of the 153 remaining articles were downloaded and read by the first two authors, who independently screened articles and applied the same inclusion and exclusion criteria as in the previous stage. The authors agreed on the inclusion of 47 articles and exclusion of 94 articles. The third author assessed the articles and solved the disagreements (*n* = 12). After discussion rounds, consensus was reached, and 59 articles were considered qualified for the review. Figure 1 shows the flowchart of the literature selection process according to the PRISMA statement.

### Data Extraction

**Fig. 2:**
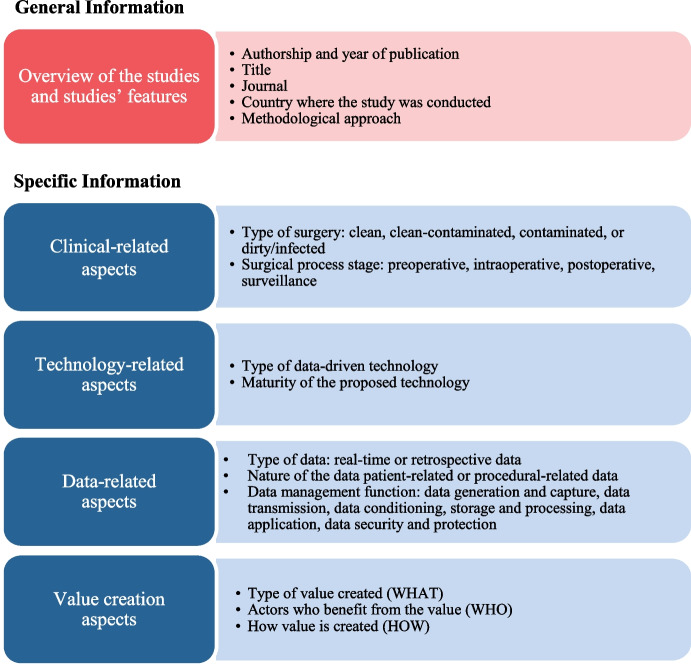
Overview of the data extraction matrix

The data were extracted manually by the first two authors and supervised by third author. To reduce human error and bias, we used a standardised data extraction spreadsheet [21]. We elaborated the data extraction categories by an iterative process driven by the study objective and based on related literature [51]. We piloted the data extraction spreadsheet on 30 randomly selected articles, refined, and modified it accordingly. The data extraction process covers general information (i.e. studies’ features) and specific information (i.e. clinic-related, technology-related, data-related, and value creation-related aspects) (see Fig. 2). Additional notes were also included by the authors on the data extraction form.

### Data Analysis

We analysed the data from a qualitative approach by using descriptive and thematic analysis techniques [21]. The descriptive analysis technique contextualises the articles in terms of general information and studies’ features, providing an overview of the characteristics of the included studies, and the clinical-related, technology-related, and data-related aspects. From a thematic analysis technique [52], we evaluated the dataset through an aggregative approach, and we manually extracted first-order codes that represent streams of value created through DDT applications in SSI prevention. We grouped the first-order codes according to their similarity, and we formulated second-order themes/categories to outline the core content of the first-order codes. Finally, we clustered the second-order themes according to the four value dimensions proposed by Smith and Colgate [44]: cost/sacrifice, functional/instrumental, experiential/hedonic, and symbolic/expressive. All the authors of the study participated in the coding process. Table 1 illustrates a sample of the coding process.

**Table 1:** Illustration (sample) of the coding process

First-order codes	Second-order themes/categories	Aggregated dimension
Monitoring cleaning and air quality in operating rooms	Improved quality and efficiency of operating room	Functional/instrumental value
Control the flow of people, materials, and other resources in operating rooms		
Sending reminders to patients and nurses via SMS	Improved communication	Experiential/hedonic value

Elaborated by the authors of the study

### Consult with Experts

To ensure methodological rigour and corroborate the research findings, three multidisciplinary focus groups and one individual interview with experts were held through a digital platform. This allowed us to review the meaning of the categories identified in the review and to discuss the implications of the study for practitioners and academic researchers [53].

The first focus group was conducted in December 2021 and aimed to present the preliminary findings of the study and to obtain additional information and perspectives on the categories and themes identified from the thematic analysis technique. Ten experts participated in this focus group: three experts in research and development of medical devices, three experts in business development for solutions in infection prevention and control, one senior researcher and one junior researcher in SSI prevention, and two senior researchers in innovation management. This focus group lasted 2 h.

The second focus group was conducted in February 2022 and aimed to review the findings of the study and analyse and refine themes and categories to attribute them a higher level of meaning. Four experts participated in this focus group: one expert in research and development of medical devices, one expert in business development for solutions in infection prevention and control, and two senior researchers in innovation management. This focus group lasted 1 h.

The third focus group was conducted in March 2022 and aimed to analyse the applicability of the findings, to assess the final categories and themes, and to discuss the implications of the study and indications of gaps and needs for future research. Four experts participated in this focus group: one expert in research and development of medical devices, one expert in business development for solutions in infection prevention and control, and two senior researchers in innovation management. This focus group lasted 1 h.

An interview with a senior infectious disease physician was conducted to discuss the research findings from a practitioner point-of-view and obtain insights on the implications of our study for infection prevention and control practices and routines. The interview was conducted in October 2022 and lasted 48 min.

## Findings

In this section, we review the 59 included articles. We organise the main findings through a descriptive analysis and a thematic analysis.

### Descriptive Analysis

Of the 59 articles reviewed, modelling/simulation is the predominant approach (28 articles) followed by experiments (12 articles). On the other hand, surveys, randomised-control trials, and quasi-experimental studies were the least adopted approaches (2 articles of each approach) (see Table 2). The data analysis matrix is shown in Online Resource 3.

**Table 2:** List of articles included in the review

Ref.	Authorship	Year of publ.	Title	Journal	Methodological approach
[54]	Kurmann et al	2011	Adverse effect of noise in the operating theatre on surgical-site infection	*British Journal of Surgery*	Observational study + survey
[55]	Nair et al	2011	Automated Electronic Reminders to Improve Redosing of Antibiotics during Surgical Cases: Comparison of Two Approaches	*Surgical Infections*	Experiment
[56]	Campillo-Gimenez et al	2013	Full-text automated detection of surgical site infections secondary to neurosurgery in Rennes, France	*MEDINFO*	Modelling/simulation
[57]	Michelson et al	2014	Assessing surgical site infection risk factors using electronic medical records and text mining	*American Journal of Infection Control*	Modelling/simulation
[58]	Zhao et al	2014	Applications for Radio-frequency Identification Technology in the Perioperative Setting	*AORN Journal*	Experiment
[59]	Du et al	2014	Real-time automatic hospital-wide surveillance of nosocomial infections and outbreaks in a large Chinese tertiary hospital	*BMC Medical Informatics and Decision Making*	Modelling/simulation
[60]	Simons et al	2014	Patient safety in the operating theatre: how A3 thinking can help reduce door movement	*International Journal for Quality in Health Care*	Experiment
[61]	Van Esbroeck et al	2014	Quantifying surgical complexity with machine learning: Looking beyond patient factors to improve surgical models	*Surgery*	Modelling/simulation
[62]	Hu et al	2015	Automated Detection of Postoperative Surgical Site Infections Using Supervised Methods with Electronic Health Record Data	*Studies in Health Technology and Informatics*	Modelling/simulation
[63]	Esser et al	2016	Reducing OR Traffic Using Education, Policy Development, and Communication Technology	*AORN Journal*	Experiment
[64]	Hwang, H	2016	Electronic wound monitoring after ambulatory breast cancer surgery: Improving patient care and satisfaction using a smart phone app	*British Columbia Medical Journal*	Observational study + survey
[65]	Gunter et al	2016	Evaluating patient usability of an image-based mobile health platform for postoperative wound monitoring	*JMIR mHealth and uHealth*	Observational study + survey
[66]	Sanger et al	2016	A Prognostic Model of Surgical Site Infection Using Daily Clinical Wound Assessment	*Journal of the American College of Surgeons*	Modelling/simulation
[67]	Sanger et al	2016	A patient-centered system in a provider-centered world: challenges of incorporating post-discharge wound data into practice	*Journal of the American Medical Informatics Association*	Descriptive study
[68]	Wiseman et al	2016	Inter-rater agreement and checklist validation for postoperative wound assessment using smartphone images in vascular surgery	*Journal of Vascular Surgery-Venous and Lymphatic Disorders*	Observational study
[69]	Ke et al	2017	Prognostics of surgical site infections using dynamic health data	*Journal of Biomedical Informatics*	Modelling/simulation
[70]	Sohn et al	2017	Detection of clinically important colorectal surgical site infection using Bayesian network	*Journal of Surgical Research*	Modelling/simulation
[71]	Sanger et al	2017	Diagnosing Surgical Site Infection Using Wound Photography: A Scenario-Based Study	*Journal of the American College of Surgeons*	Survey
[72]	Eskildsen et al	2017	The effect of a door alarm on operating room traffic during total joint arthroplasty	*Orthopedics*	Experiment
[73]	Weller et al	2017	Leveraging electronic health records for predictive modeling of post-surgical complications	*Statistical Methods in Medical Research*	Modelling/simulation
[74]	Falen et al	2017	Using the Electronic Health Record Data in Real Time and Predictive Analytics to Prevent Hospital-Acquired Postoperative/Surgical Site Infections	*The Health Care Manager*	Descriptive study
[75]	Gunter et al	2018	Feasibility of an Image-Based Mobile Health Protocol for Postoperative Wound Monitoring	*Journal of the American College of Surgeons*	Survey
[76]	Armellino et al	2018	Remote Video Auditing to Verify OR Cleaning: A Quality Improvement Project	*AORN Journal*	Experiment
[77]	Ribed et al	2018	Improving surgical antibiotic prophylaxis adherence and reducing hospital readmissions: A bundle of interventions including health information technologies	*European Journal of Hospital Pharmacy*	Quasi-experimental study
[78]	Canty and St George	2018	Development of a surgical site infection surveillance programme in a Scottish neurosurgical unit	*International Journal of Health Governance*	Descriptive study
[79]	Broman et al	2018	Evaluation of Wound Photography for Remote Postoperative Assessment of Surgical Site Infections	*JAMA Surgery*	Quasi-experimental study
[80]	Lu et al	2018	Accessible Communication Tools for Surgical Site Infection Monitoring and Prevention in Joint Reconstruction: Feasibility Study	*JMIR Perioperative Medicine*	Experiment
[81]	Rosner et al	2018	Accuracy of Internet-Based Patient Self-Report of Postdischarge Health Care Utilization and Complications Following Orthopedic Procedures: Observational Cohort Study	*Journal of Medical Internet Research*	Observational study + survey
[82]	Weiser et al	2018	The Effect of Door Opening on Positive Pressure and Airflow in Operating Rooms	*Journal of The American Academy of Orthopaedic Surgeons*	Experiment
[83]	Bartz-Kurycki et al	2018	Enhanced neonatal surgical site infection prediction model utilizing statistically and clinically significant variables in combination with a machine learning algorithm	*The American Journal of Surgery*	Modelling/simulation
[84]	Mousa et al	2019	Results of Telehealth Electronic Monitoring for Post Discharge Complications and Surgical Site Infections following Arterial Revascularization with Groin Incision	*Annals of Vascular Surgery*	Experiment
[85]	Childs et al	2019	The surgical wound in infrared: thermographic profiles and early stage test-accuracy to predict surgical site infection in obese women during the first 30 days after caesarean section	*Antimicrobial Resistance and Infection Control*	Observational study
[86]	Hsu et al	2019	Chronic wound assessment and infection detection method	*BMC Medical Informatics and Decision Making*	Modelling/simulation
[87]	Birgand et al	2019	Motion-capture system to assess intraoperative staff movements and door openings: Impact on surrogates of the infectious risk in surgery	*Infection Control and Hospital Epidemiology*	Observational study
[88]	Zhang et al	2019	Monitoring Surgical Incision Sites in Orthopedic Patients Using an Online Physician–Patient Messaging Platform	*Journal of Arthroplasty*	Descriptive study
[89]	Shen et al	2019	Detection of Surgical Site Infection Utilizing Automated Feature Generation in Clinical Notes	*Journal of Healthcare Informatics Research*	Modelling/simulation
[90]	Gowd et al	2019	Construct validation of machine learning in the prediction of short-term postoperative complications following total shoulder arthroplasty	*Journal of Shoulder and Elbow Surgery*	Modelling/simulation
[91]	Haskins et al	2019	Development and Validation of the Ventral Hernia Repair Outcomes Reporting App for Clinician and Patient Engagement (ORACLE)	*Journal of the American College of Surgeons*	Modelling/simulation
[92]	Tunthanathip et al	2019	Machine learning applications for the prediction of surgical site infection in neurological operations	*Neurosurgical Focus*	Modelling/simulation
[93]	da Silva et al	2019	Predicting the occurrence of surgical site infections using text mining and machine learning	*PLoS One*	Modelling/simulation
[94]	Azevedo-Coste et al	2019	Tracking Clinical Staff Behaviors in an Operating Room	*Sensors*	Experiment
[95]	Azimi et al	2020	Post-Operative Infection Prediction and Risk Factor Analysis in Colorectal Surgery Using Data Mining Techniques: A Pilot Study	*Surgical Infections*	Modelling/simulation
[96]	Bucher et al	2020	Portable Automated Surveillance of Surgical Site Infections Using Natural Language Processing: Development and Validation	*Annals of Surgery*	Modelling/simulation
[97]	Chang et al	2020	Deep Learning-Based Risk Model for Best Management of Closed Groin Incisions After Vascular Surgery	*Journal of Surgical Research*	Modelling/simulation
[98]	Chen et al	2020	Artificial Intelligence-Based Multimodal Risk Assessment Model for Surgical Site Infection (AMRAMS): Development and Validation Study	*JMIR Medical Informatics*	Modelling/simulation
[99]	Ciofi Degli Atti et al	2020	Developing a Surgical Site Infection Surveillance System Based on Hospital Unstructured Clinical Notes and Text Mining	*Surgical Infections*	Modelling/simulation
[100]	Hopkins et al	2020	Using artificial intelligence (AI) to predict postoperative surgical site infection: A retrospective cohort of 4046 posterior spinal fusions	*Clinical Neurology and Neurosurgery*	Modelling/simulation
[101]	Karhade et al	2020	Can natural language processing provide accurate, automated reporting of wound infection requiring reoperation after lumbar discectomy?	*Spine Journal*	Modelling/simulation
[102]	Park et al	2020	Detection of Bacteremia in Surgical In-Patients Using Recurrent Neural Network Based on Time Series Records: Development and Validation Study	*Journal of Medical Internet Research*	Observational study
[103]	Park et al	2020	The value of preoperative spirometry testing for predicting postoperative risk in upper abdominal and thoracic surgery assessed using big-data analysis	*Journal of Thoracic Disease*	Modelling/simulation
[104]	Schlund et al	2020	Value of an interactive phone application in an established enhanced recovery program	*International Journal of Colorectal Disease*	Experiment
[105]	van Niekerk et al	2020	Risk factors for surgical site infections using a data-driven approach	*PLoS One*	Descriptive study
[106]	Formeister et al	2020	Machine Learning for Predicting Complications in Head and Neck Microvascular Free Tissue Transfer	*The Laryngoscope*	Modelling/simulation
[107]	Elhage et al	2021	Development and Validation of Image-Based Deep Learning Models to Predict Surgical Complexity and Complications in Abdominal Wall Reconstruction	*JAMA Surgery*	Modelling/simulation
[108]	McLean et al	2021	Remote diagnosis of surgical-site infection using a mobile digital intervention: a randomised controlled trial in emergency surgery patients	*NPJ Digital Medicine*	Randomised clinical trial
[109]	Petrosyan et al	2021	Predicting postoperative surgical site infection with administrative data: a random forests algorithm	*BMC Medical Research Methodology*	Modelling/simulation
[110]	Shahroudi et al	2021	Quality improvement through lean A3 method for foot traffic in operating room	*Perioperative Care and Operating Room Management*	Experiment
[111]	Wang et al	2021	Effect of Nursing Intervention in the Operating Room Based on Simple Virtual Reality Augmented Technology on Preventing Gastrointestinal Surgical Incision Infection	*Journal of Healthcare Engineering*	Randomised clinical trial
[112]	Zhu et al	2021	Applying Machine Learning Across Sites: External Validation of a Surgical Site Infection Detection Algorithm	*Journal of the American College of Surgeons*	Modelling/simulation

Elaborated by the authors of the study

**Fig. 3:**
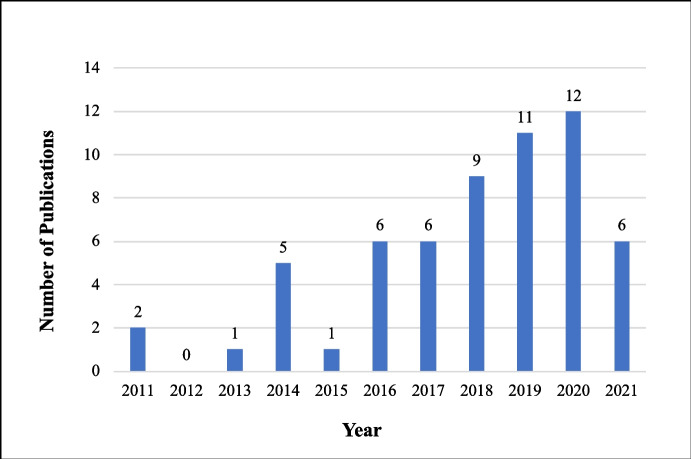
Distribution of articles by year of publication

The distribution of the reviewed articles per year of publication illustrates that from 2011 to 2015, only 9 articles were published (see Fig. 3). In contrast, there was an exponential growth in the number of articles published between 2016 and 2020. In this period, 44 articles were published, which suggests the growing interest in the subject. 2020 was the year with the highest number of articles published in the period (12 articles), which is equivalent to twice the number of publications in 2016 (6 articles).

Drawing on the distribution of articles by country (see Table 3), most of the studies were conducted in the USA (35 articles), which can be explained by two factors: first, the high level of public and private investment in medical technology research and development in the USA, which accelerates the emergence of healthcare innovations [113], and second, the US healthcare industry is highly competitive and companies in this industry seek for the development of new and more profitable solutions that enable the delivery of high-quality and more cost-effective services [114].

**Table 3:** Distribution of articles by country where the studies were conducted

Country	Number of publications	%
USA	35	59
China	4	7
UK	3	5
France	3	5
The Netherlands	3	5
Canada	2	3
South Korea	2	3
Others (only with 1 study)	7	12
Total	59	100

Elaborated by the authors of the study

**Fig. 4:**
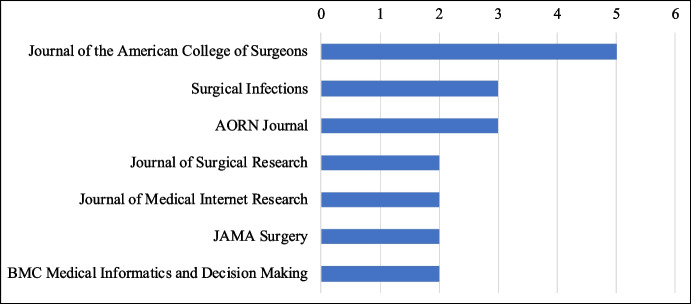
Top journals with at least two publications included in the review

Although SSI prevention is recognised as a multidisciplinary field [115], most of the articles included in this review were published in health sciences journals and few other publications in health informatics journals (see Fig. 4). No published articles were found in innovation management, organisational studies, and information systems journals.

#### Clinical-Related Aspects

The clinical-related aspects include the type of surgery and the patient care stages where DDTs are applied (see Table 4).

**Table 4:** Clinical-related aspects

Type of surgery	Patient care stage
Clean: [56] [57] [60] [63] [64] [65] [68] [72] [75] [77] [80–82] [84] [87] [88] [90] [92] [94] [97] [100] [101] [106] *Total: 23 articles*	Preoperative: [61] [70] [73] [77] [80] [83] [84] [88] [92] [98] [104] [105] [106] [107] *Total: 14 articles*
Clean-contaminated: [54] [66] [69] [70] [71] [73] [79] [85] [89] [91] [95] [103] [104] [107] [108] [111] *Total: 16 articles*	Intraoperative: [54] [58] [60] [63] [70] [72] [76] [77] [82] [87] [92] [94] [110] [111] *Total: 14 articles*
Contaminated: -	Postoperative: [55] [64–70] [73–75] [77] [79] [80] [81] [83–86] [88] [90–92] [98] [104–109] *Total: 30 articles*
Dirty: -	Surveillance: [56] [57] [62] [71] [78] [79] [89] [93] [95–97] [99–103] [112] *Total: 17 articles*
Non specified: [55] [58] [59] [61] [62] [67] [74] [76] [78] [83] [86] [93] [96] [98] [99] [102] [105] [109] [110] [112] *Total: 20 articles*	

Elaborated by the authors of the study

Studies describe technologies applied to manage data originated from clean, clean-contaminated, contaminated, and dirty surgeries [26]. Of the 59 reviewed articles, 23 outline the use of DDTs in the prevention of SSIs in clean surgeries, such as orthopaedic surgeries [63] and neurosurgeries [92]. Sixteen articles described prevention of clean-contaminated surgeries such as gastrointestinal surgeries [111] and colorectal procedures [104]. No studies were found that mentioned the application of DDTs to prevent contaminated or dirty surgeries. This is not surprising given the fact that patients undergoing contaminated and dirty surgeries are more likely to develop an SSI, mostly due to endogenous risk factors that are difficult to control [116, 117].

The patient care stages are associated with the risk factors of acquiring SSIs in different stages of the surgical process. In this review, we classified DDTs tailored to manage data in SSI prevention in the preoperative, intraoperative, postoperative, and surveillance stages [28]. In total, 30 articles focus on the postoperative stage. DDT applications at this stage are designed mainly to facilitate the monitoring of surgical wounds healing by assessing wounds pictures shared via smartphone applications [64, 75] and to allow clinicians to inform patients of postoperative procedures and antibiotic consumption via electronic reminders [55].

Fourteen articles addressed the use of DDTs in the preoperative stage. Most of DDT applications in this stage are aimed to predict SSI occurrence based on patient clinical data (e.g. age and eating habits) and on the complexity of surgical procedures to be performed. For example, van Esbroeck et al. [61] described the use of machine learning to successfully predict the outcomes of mortality, morbidity, SSI rates, and other complications within 30 days of surgery.

Fourteen articles addressed the use of DDTs in the intraoperative stage, mainly focusing on the identification and mitigation of SSI risks related to behavioural and environment aspects. For example, Birgand et al. [87] described how a motion-capture system based on cameras and smart sensors was used to monitor operating room traffic and predict SSI occurrence. The system analysed how the number of door openings during surgical procedures increase the number of particles in the air, increasing the risks of wound contamination. Similarly, Zhao et al. [58] assessed the quality of the surgical procedures by monitoring the usage of personal protective equipment and hand disinfection practices by surgical teams in operating rooms by using radiofrequency sensors.

Seventeen articles described the use of DDTs such as machine learning systems in SSI surveillance. In this stage, the use of machine learning algorithms helps clinicians and infection preventionists to analyse large amount of data from hospital databases, allowing rapid and accurate identification SSI trends and detect potential ongoing outbreaks [59]. In some cases, artificial intelligence-based surveillance systems can achieve up to 100% accuracy in identifying SSIs [57, 99].

#### Technology-Related Aspects

The technology-related aspects comprise the type of technology used and the maturity of the proposed technology (see Table 5).

**Table 5:** Technology-related aspects

Main type of DDT evidenced	Inferred maturity of the technology
Big Data analytics*: [103] *Total: 1 article*	TRL1: [54] [56] [57] [60] [63] [68] [71] [76] [79] [82] [83] [86] [87] [89] [90] [92] [93] [95] [98] [100–103] [105–107] [109–112] *Total: 31 articles*
Data mining*: [56] [57] [59] [95] [99] *Total: 5 articles*	TRL2: [78] *Total: 1 article*
Deep learning: [97] [102] [107] *Total: 3 articles*	TRL3: [55] [72] [73] [81] [84] [99] *Total: 6 articles*
Digital cameras: [71] [76] *Total: 2 articles*	TRL4: [70] [77] [80] [91] [97] *Total: 5 articles*
Internet and software applications: [55] [74] [77–81] *Total: 7 articles*	TRL5: [62] *Total: 1 article*
Machine learning: [61] [62] [66] [69] [70] [73] [83] [86] [89] [90] [92] [93] [96] [98] [100] [101] [105] [106] [109] [112] *Total: 20 articles*	TRL6: [61] [66] [69] [74] [75] [96] [108] *Total: 7 articles*
Smartphone applications: [64] [65] [67] [68] [75] [84] [88] [91] [104] [108] *Total: 10 articles*	TRL7: -
Smart sensors: [54] [58] [60] [63] [72] [82] [85] [87] [94] [110] *Total: 10 articles*	TRL8: [58] [94] *Total: 2 articles*
Virtual reality: [111] *Total: 1 article*	TRL9: [59] [64] [65] [67] [88] [104] *Total: 6 articles*

^*^Although Big Data analytics and data mining are not technologies but techniques and methods, they are essential to understanding the technological aspects of value creation by DDTs

Elaborated by the authors of the study

The most common DDTs were machine learning-based systems (20 articles), followed by smartphone applications (10 articles) and smart sensors (10 articles), while the least mentioned DDTs were virtual reality (1 article) and Big Data analytics (1 article).

We categorised the DDTs according to the maturity of the technology proposed. The level of the maturity is defined by the technology readiness assessment and can vary on a scale from technology readiness level (TRL) 1 to 9, where 1 means that the research findings are started to being translated into more applied research, while 9 means that the technology has succeed in being applied [118]. Out of the 59 reviewed articles, 31 described methods or techniques based on DDTs to prevent SSIs, without developing or launching any new technology. For example, Armellino et al. [76] developed an auditing method based on remote monitoring to assess compliance with good cleaning practices in operating rooms, while Campillo-Gimenez et al. [56] proposed a model to compare the advantages of data mining techniques with manual surveillance methods. Therefore, these solutions can be classified in the TRL1 level, where basic principles are observed and reported through primary research.

Six articles described DDTs in the TRL9 level. For example, Gunter et al. [65] reported the development and testing of an image-based smartphone application for post discharge surgical wound monitoring. The smartphone application was launched in the market and made available for patients and healthcare practitioners. Similarly, Du et al. [59] described the development of an automated surveillance system to monitor infections and outbreaks. The system has proven its efficiency after 4 years of development and testing and has already been adopted in several hospitals in China.

#### Data-Related Aspects

Data-related aspects refer to the characteristics of data used and how the data is managed for SSI prevention purposes. We classified the articles according to the type of data, nature of data, and data management function (see Table 6). Similar to Khazaei et al. [119], we classified the articles according to the type of data used, i.e. real-time or retrospective data.

**Table 6:** Data-related aspects

Type of data used	Nature of data	Data management function
Mainly real-time data: [55] [58–60] [63–69] [72] [74–78] [80–82] [84] [85] [87] [88] [94] [104] [108] [110] [111] *Total: 29 articles*	Mainly patient-related data: [55–57] [59] [62] [64–71] [75] [79–81] [84–86] [89–92] [95] [98] [99] [102] [107] [108] [109] [112] *Total: 32 articles*	Data generation or capture: [54–60] [63–73] [75] [76] [81–89] [91] [94] [96–99] [104] [108] [110] [111] *Total: 39 articles*
Mainly retrospective data: [54] [56] [57] [61] [62] [70] [71] [73] [79] [83] [86] [89] [90–93] [95–103] [105–107] [109] [112] *Total: 30 articles*	Mainly procedure-related data: [54] [58] [60] [63] [72] [76] [82] [87] [94] [110] [111] *Total: 11 articles*	Data transmission: [58] [60] [63–68] [72] [75] [76] [80–82] [84] [87] [88] [91] [94] [104] [108] [110] [111] *Total: 23 articles*
	Combination of patient-related and procedure-related data: [61] [73] [74] [77] [78] [83] [88] [93] [96] [97] [100] [101] [103–106] *Total: 16 articles*	Data storage, conditioning or processing: [56–59] [60–67] [69–75] [77–79] [81] [83] [84] [86] [88–112] *Total: 51 articles*
		Data application: [58] [60] [61] [63] [65] [67] [69] [70] [72] [74] [77] [89] [94] [103] [104] [108–111] *Total: 19 articles*
		Data security or protection: [65] [68] [80] [81] [94] [97] [98] [104] [108] *Total: 9 articles*

Elaborated by the authors of the study

Retrospective data is the most common type of data present in the reviewed articles (30 articles). Although 29 articles reported the use of real-time data in SSI prevention, most of them described the collection of real-time data, but with a retrospective data analysis. For example, studies described the monitoring of surgical team behaviour in operating rooms in real time [94, 110]. In these studies, the data is converted and stored, and the analyses are performed offline, i.e. several days after the surgery. One of the exceptions is the study by Falen et al. [74] that describes the collection and analysis of data in real time. The article proposed the collection and interpretation of electronic medical record data in real time to prospectively identify patients’ needs of early interventions during hospital stay.

We classified the articles according to the nature of the SSI risk factors represented by the data, i.e. patient-related data (endogenous risk factors) and procedure-related data (external risk factors) [22]. Out of 59 articles reviewed, 32 articles described the use of patient-related data, 11 articles described the use of procedure-related data, and 16 articles reported the use of patient-related and procedure-related data combined. Patient-related data is mainly obtained from secondary sources such as medical records [62] or national electronic databases [91] but can also be generated and shared by the patients or patients’ family members after patient discharge [67, 99].

Procedure-related data refers to the data that is directly or indirectly related to the healthcare staff behaviour and can influence in the SSI incidence. Direct behavioural data is obtained by the monitoring of routines, such as cleaning procedures [76], duration of surgical procedures [58], and staff movements in the operating room [94]. Indirect behavioural data is obtained by the monitoring of environmental conditions that are affected by human actions, such as the air quality in the operating room [82] and the level of noise during surgical procedures [54].

We classified the articles according to the purpose of the DDTs in terms of data management function, i.e. data generation and capture, data transmission, data conditioning, storage and processing, data application, and data security and protection [7, 8].

Thirty-nine articles mentioned the use of DDTs to generate or capture data. For example, Childs et al. [85] described the use of sensors to generate data related to surgical wounds’ temperature, while Hsu et al. [86] and Sanger et al. [71] described the use of digital cameras integrated in smartphones to obtain pictures of surgical wounds. Fifty-one articles mentioned the use of DDTs used in storage, conditioning, or processing data. They mainly described the use of integrated information systems that enable the storage of electronic medical reports data and the use of artificial intelligence-based algorithms that standardise, prepare, and analyse the data [90, 95, 100].

Nineteen articles reported the use of DDTs for data application. For example, the study of Eskildsen et al. [72] described a system that reduces the number and frequency of door openings in operating rooms through the application of data collected by sensors. The system monitors door openings and time left ajar during surgical procedures by using an electronic sensor. Integrated to the sensor, an audible alarm was placed to sound continuously when the door was ajar, which promoted a significant decrease in the number of door openings per surgery.

Nine articles described data security or protection issues, but few of them provide details on how DDTs are used to achieve it. For example, the study of Gunter et al. [65] reported the use of a smartphone application by patients with the function of capturing pictures of surgical wounds and sharing them with surgeons and nurses. To ensure data protection, patients are encouraged not to send pictures that included identifying marks or their face. The application does not allow the storage of pictures on the mobile phone itself and only can be used to submit information, not retrieve it. Additionally, a passcode is used to secure and encrypt the device.

### Thematic Analysis

In this section, we present the categories of value identified from the reviewed articles and the classification according to the four value dimensions proposed by Smith and Colgate [44]: cost/sacrifice, functional/instrumental, experiential/hedonic, and symbolic/expressive.

#### Cost/Sacrifice Value

Cost/sacrifice value is described by the perceived benefits from the use of a DDT in SSI prevention, in relation to the efforts to use, buy, or own the DDT. Most of the reviewed studies indicated improvements in the quality of healthcare and *cost reduction* as outcomes [58, 97, 104], but few studies presented evidence on how different actors perceive the cost-benefits relationship. For example, Nair et al. [55] reported the implementation of an electronic reminder system to support anaesthesia teams in performing timely administration of antibiotic re-doses, which helps to minimise the risk of SSIs. The software has proven its cost-effectiveness by ensuring a re-dose success rate of about 95%, despite the costs for the development and maintenance of the system, estimated in $40,000 and 6 months and $10,000 per year, respectively [55].

Gunter et al. [65] evaluated the average usability of an image-based mobile application for postoperative wound monitoring among patients. Although almost 45% of the patients faced problems in completing the training sessions and faced challenges while using the application, 83.3% of them were satisfied with the solution. Yet, 81.8% of the images taken by the patients were good enough for physicians to monitor surgical wounds and come up with precise diagnosis [65]. Similarly, Zhang et al. [88] estimated a decrease in 14% of unnecessary clinic visits with the use of an online application for physician–patient messaging, despite the non-specified financial costs inherent to the implementation of the solution.

#### Functional/Instrumental Value

Functional/instrumental value is concerned with the technical features of the DDTs and the reliability and accuracy of the data collected and analysed. We identified four types of value created: (i) *speed-up patient recovery*, (ii) *timely and accurate diagnosis*, (iii) *improved quality and efficiency of operating room*, and (iv) *risk prediction*.

A range of studies reported the use of smartphone applications to provide guidance to patients on basic care and hygiene practices for the treatment of operative wounds [64, 67, 68, 75, 84]. Physicians and nurses can monitor the wound healing process, send follow-up messages and reminders about antibiotic administration, and answer patients’ questions about treatments, ultimately helping to *speed-up patient recovery*. The findings of Hwang [64] indicate that 95% of the patients who used smartphone applications for postoperative monitoring said that their recovery process was faster and safer. The study revealed that only 3% of patients who used smartphone applications for postoperative monitoring needed unscheduled visit to emergency departments, while 22% of patients in conventional follow-up groups needed unscheduled care [64].

The timely identification of ongoing SSIs is crucial so that patients can receive early treatment to avoid the worsening of the clinical condition and perhaps causing cross-transmission of infections. Studies described the application of DDTs to obtain *timely and accurate diagnosis* on SSIs [56, 96]. For example, studies described the development of surveillance systems based on machine learning to collect and analyse patient-related data to identify potential SSI occurrences with high level of accuracy and in real time [69]. To increase the sensitivity of the algorithms, the systems combined multiple sources of data, such as microbiological reports, antibiotic usage reports, and imaging reports. As a result, the algorithms can rapidly detect wound colonisation, SSI clusters, and outbreaks during the early stages, thus allowing nurses and clinicians to implement prevention and control strategies with more efficacy [59, 108].

Studies described DDT applications to *improve the quality and efficiency of operating rooms* as an important aspect for SSI prevention. In this regard, smart sensors allow surgical teams to monitor and control environmental conditions that are associated with SSI. For example, studies reported the use of sensors and other DDTs to monitor and reduce the number of door openings and people traffic in operating rooms, thus helping with maintaining appropriate levels of pressure, airflow, and noise [54, 60, 63, 72]. Smart sensors are also used to limit people’s access to operating rooms [58] and reduce the number of movements by staff during surgeries [94], thus helping to maintain the sterile environment and to make the flow of personnel and materials more effective.

Preventing SSI occurrence relies on the identification and mitigation of endogenous and exogenous risks associated with the likelihood of a patient developing an SSI. In this regard, studies reported the use of machine learning algorithms to *predict risk* factors by analysing data related to patients’ health conditions (e.g. age, alcohol consumption, comorbidities) and complexity of surgical procedures [57, 61, 105]. As hospital databases often contain large amount of incomplete and unstructured data, machine learning models can be designed to decipher complex relationships between variables and perform statistical analyses beyond the scope of human capability [83]. The study of Falen et al. [74] described the assessment of multiple risk factors in the real time, where machine learning algorithms estimate the probability of SSI occurrence by integrating data from hospital databases with patients’ follow-up reports in the postoperative stage. Similarly, Ke et al. [69] proposed an algorithm that predicts SSI risks by analysing evolving clinical variables, also suggesting that machine learning models that integrates dynamic data present better prediction accuracy, thus being more reliable.

#### Experiential/Hedonic Value

Experiential/hedonic value is concerned to positive feelings, sensations, and emotions that patients, healthcare practitioners, and managers experience when using DDTs. It is also associated to the benefits of social interactions and knowledge creation. Regarding this dimension, we identified three types of value created: (i) *convenience and comfort*, (ii) *improved communication*, and (iii) *shared decision-making*.

DDTs in general leverage the automation of processes and allow the execution and delivery of health services with reliability and comfort. In this sense, we assume that all reviewed articles directly or indirectly address the use of DDTs to enhance *convenience and comfort*. Here, however, we only include the articles that explicitly evidence the creation of this type of value. For example, studies portray the use of smartphone applications as an important tool to allow remote postoperative care. Broman et al. [79] reported that surgeons prefer using smartphone applications for sending and receiving photos of patient wounds rather than in-person clinical assessments. Surgeons also claim that remote care allows more flexibility with time management and therefore enhances efficiency, as the duration of remote consultations is slightly shorter than the duration of in-person consultations [79].

Studies suggest that patients and their family members are enthusiastic about using smartphone applications for remote care, as it reduces or eventually eliminates the need for patients to return to the hospital settings [75, 79], thus avoiding being expose to innumerable infectious bacteria and viruses. In addition, it alleviates the burden and costs of unnecessary travels for clinical visits, which is highly beneficial, as patients are usually more vulnerable during the post discharge. In this sense, remote care, in addition to being more convenient and cheaper, is also safer [75] and helps to increase patient satisfaction with the healthcare received [64].

By nature, DDTs are meant to make communication easier, faster, and more efficient [7]. In the context of SSI prevention, DDTs directly or indirectly foster *improved communication* between healthcare practitioners and patients. In our analysis, however, we consider the studies that explicitly indicate better communication flow between actors. For example, Lu et al. [80] described the development and implementation of two software applications that generate automated text messages and phone calls with the aim of regularly reminding patients about the preoperatory procedures and monitoring signs and symptoms of SSIs postoperatively. As a result, patients felt more confident by receiving medical advice on a regular basis, while nurses and surgeons are efficient with monitoring patients’ outcomes based on auto-generated reports, without an increased workload [80]. Similarly, other studies reported that patients appreciate the direct connection with nurses and physicians via smartphone applications, demonstrating more interest in interacting with these professionals and asking questions about wound treatments, medications, and medical appointments [75, 84].

Over the surgical stages, healthcare practitioners from multiple specialities are challenged to work collaboratively and combine their competences to alleviate bias and reduce SSI risk factors [120]. In this context, DDTs serve as digital platforms where practitioners can access and share patient-related data and discuss about diagnoses and patients’ conditions, thus leading to *shared decision-making*. For example, Falen et al. [74] described a software application that can automatically pull patient-related data from various sources and perform simultaneous analysis on SSI risk factors. Through this software, nurses and physicians receive real-time notifications about laboratory results, patient vital signs, current medications, past procedures, and coexisting and comorbid conditions. The software predicts and ranks SSI risk factors and denotes the likelihood that an SSI will occur, ultimately helping nurses and physicians to share decision treatments and other early interventions [74].

#### Symbolic/Expressive Value

Symbolic/expressive value refers to when the actors involved in SSI prevention associate DDTs with more abstract or symbolic meanings, here represented by *patient empowerment*.

Although some reviewed studies address the efforts of patients in reducing SSI risks [67, 81], few studies provide details on how DDTs effectively contribute to make them feel protagonists in SSI prevention routines. For example, Haskins et al. [91] reported the development and validation of a digital platform based on software and smartphone applications that allow patients to do virtual simulations to estimate SSI risks based on their own conditions at the preoperative and postoperative stages. Similarly, McLean et al. [108] reported that the use of a smartphone application for remote wound assessment helped patients to be more engaged in communicating with physicians and nurses and encouraged them to lead their own postoperative recovery.

In Table 7, we present an overview on the created value categories and dimensions. By following the value space logic proposed by Lindman et al. [47], we also indicate the actors who mainly benefit from the created value and identify the predominant DDTs and the key activities that enable them as a source of value creation in SSI prevention.

**Table 7:** Overview of value creation aspects

Value dimension	Value created—WHAT	Main actors—WHO	Predominant DDT	Activities—HOW
Cost/sacrifice value	Cost reduction	Hospitals	Smartphone and software applications	Smartphone and software applications help to optimise the use of resources and settings (e.g. operating rooms) and support decision-makers with planning the distribution of time and resources
Functional/instrumental value	Speed-up patient recovery	Patients, nurses, physicians	Smartphone applications	With smartphone applications, patients can get proper advice on how to treat wounds and take antibiotics and get some tips to help with the recovery process
	Timely and accurate diagnosis	Physicians, nurses	Machine learning	Algorithms can detect potential SSIs based on patient-related data obtained from multiple with high accuracy
	Improved quality and efficiency of operating room	Surgical teams	Smart sensors	Sensors allow the monitoring of environmental conditions of operating rooms, e.g. temperature, cleaning, air quality and traffic of people, materials, and other resources
	Risk prediction	Surgeons, infection prevention, and control teams	Machine learning	Machine learning models can predict SSI risks based on the analysis of large datasets from hospital databases, patient records, and medical notes
Experiential/hedonic value	Convenience and comfort	Surgeons, patients, and patients’ families	Smartphone applications	With smartphone applications, patients can receive remote treatment, while avoid unnecessary hospital visits and readmissions. Smartphone applications also gives more flexibility to surgeons with time management
	Improved communication	Surgeons, nurses, patients	Smartphone and software and applications	Smartphone and software applications allow healthcare practitioners and patients to share relevant information about appropriate health conditions and preparation for surgeries and patient recovery process
	Shared decision-making	Surgeons, nurses	Software applications	Software applications enable nurses and physicians to share information about patients, procedures, and treatments and to make joint decisions of early interventions to mitigate SSI risks
Symbolic/expressive value	Patient empowerment	Patients	Smartphone applications	With smartphone applications, patients take an active role in the prevention of SSIs and lead their own recovery process

Elaborated by the authors of the study

## Conclusions

In this systematic review, we explored how DDTs enable value creation in the prevention of SSIs. DDTs include, among others, cloud computing, Big Data analytics, and artificial intelligence that enable digital, networked, automated, and intelligent processes [5, 6]. We follow the PRISMA protocol to review empirical studies retrieved from the databases Scopus, Web of Science, MEDLINE, ProQuest, PubMed, ABI/Inform Global, and Cochrane. In total, 59 articles published between 2011 and November 2021 were reviewed and analysed from descriptive and thematic analysis techniques. There was a substantial growth in the number of articles published between 2016 and 2020, which suggests an increasing interest in applications of DDTs for the prevention of SSIs. Most of the articles are published in health sciences and health informatics journals, predominantly with a modelling/simulation approach. Out of 59 articles analysed, 35 (59%) were conducted in the USA.

Our study provides an overview on the clinical, technological, and data-related aspects associated to value creation enabled by DDTs in SSI prevention. Regarding the clinical aspects, most of the reviewed articles mention DDT applications aimed to prevent infections in the postoperative and surveillance stages. This is in line with the fact that 32 out of 59 articles address the use of patient-related data, which suggests that DDTs are primarily designed to monitor patient recovery, to support SSI diagnosis, and to provide data on postoperative treatments and routines. Furthermore, 23 articles show the use of DDTs in the prevention of SSIs in clean surgeries, while no studies were found about dirty or infected surgeries. In fact, almost half of SSIs in clean surgery are preventable [121], which justifies the focus on technological solutions for this type of surgery. However, it is noteworthy that SSIs in clean surgeries represent less than 2% of all SSIs, while SSIs in dirty surgeries represent more than 40% of occurrences [27].

Regarding technology-related aspects, our findings show that machine learning is reported in 21 articles, which is in line with previous reviews that have evidenced that machine learning has become one of the most popular DDTs, as it allows to identify risk factors and trends that would be difficult for human operators to perceive [8, 14]. Our findings indicate that in 31 out of 59 articles, the technologies are in the first stage of the technology readiness scale (TRL1), which means that the research findings are started to being translated into more applied research, thus denoting a growing interest in the development of innovative solutions for the prevention of SSI and the potential emergence of new DDTs.

Regarding the data-related aspects, our findings indicate that 29 articles are focused on the use of real-time data, while 30 articles are focused on the use of retrospective data. This is problematic, as prior literature suggests that the combination of retrospective and real-time data produces more reliable results and allows the identification of SSI risk factors with more accuracy and speed [122]. In relation to the nature of the data, 32 articles report DDTs that rely mainly on patient-related data, while 11 articles include procedure-related data. The reviewed articles indicate that DDTs are mainly used to store, condition, and process data (51 articles), while few studies provide information on how to ensure data security or protection while using DDTs (9 articles). Privacy and data security issues are often mentioned as key barriers for the implementation of new technologies in healthcare, and scholars claim that these issues should be among the top priorities for academics, companies, and practitioners [4, 7].

Following the value space logic [47], we linked the value categories and value dimensions with the predominant DDTs and the key actors and activities that enable value creation in SSI prevention. We classified the value created by DDTs in 9 categories, organised according to 4 value dimensions: *cost/sacrifice value*, *functional/instrumental value*, *experiential/hedonic value*, and *symbolic/expressive* value.

Our findings suggest that studies address the creation of instrumental value, as many of them deals with functional aspects of technologies, focusing on the technical features and on the accuracy and reliability of collected and analysed data. Physicians and nurses are the actors who directly benefit. While most studies report improvements in patient care by reducing SSI risk factors, few provide data on cost-effectiveness of DDTs, especially in terms of financial and non-financial impacts for patients and healthcare practitioners. The few studies that explicitly address cost–benefit relationships focus on the perspective of hospitals, paying scant attention to how other actors perceive the benefits and efforts involved in buying and using DDTs. On the other hand, studies that outline the creation of symbolic value are mostly focused on patient empowerment, thus overlooking personal and social aspects of other individuals and groups.

### Implications

Our study aggregates and classified relevant knowledge about DDT applications in the prevention of SSIs from a multi-actor perspective and provides an overview of the clinical, technological, and data-related aspects associated to the use of various DDTs in SSI prevention.

Our study analyses the categories of value that are enabled by DDT applications, relate them to the actors who mainly benefit from DDTs, and outline the activities by how the value created is perceived. Empirical evidence suggests that technological innovations in healthcare are often rejected or abandoned by individuals and groups because they are unable to perceive how they can benefit from them [123]. We believe that our study offers insights to hospitals and healthcare practitioners and managers on how DDTs can enable value creation and to whom the benefits must be communicated, which can help to reduce resistance to technology acceptance [124, 125]. Our findings can help medical technology companies to identify and structure new opportunities to generate benefits for users through the development of new technologies or the integration of new features in current products and services that can maximise the perception of value created.

### Limitations of the Study

Our study is subject to limitations. First, the publication period only covers 10 years. However, given the rapid advance of DDTs, we do not consider this to be a serious issue. A potentially greater limitation is that the study does not include unpublished data and grey literature. However, we expect that overlooked studies will soon be published to the extent they are essential.

Second, our study focuses on technology and how they can create value but does not deal with new services or product-service systems. A product-service system approach is grounded on the idea that value results from the experiences that customers and end-users have with combined sets of products, services, and software [126]. In this context, value is co-created inside-hospital or outside-hospital, and in offline or online settings, driven by dynamic interactions between patients, healthcare practitioners and products, services, and software [127]. Although we identified elements that illustrate the interaction between multiple actors through digital platforms (e.g. the use of smartphone applications for remote care), the source material is limited to the traditional view of value creation of the relation of the technology vs single type of user.

Third, our study provides a broad overview on multiple aspects associated to value creation enabled by DDTs in SSI prevention, but our findings are limited by the study design and scope. We reviewed a small sample of 59 articles with heterogeneous approaches and great diversity of contexts of DDT applications, which limits the generalizability of our findings. Furthermore, as we did not perform a quality assessment of the reviewed articles, it is not possible to estimate the extent to which our findings may be transferable to other situations and settings.

### Future Research

Based on the analysis of the 59 studies reviewed and on the limitations of our study, we identified four opportunities for future research:

- **Value co-creation and product-service systems**: Given the advances on the literature towards value-based care and grounded on the participation of multiple actors in the joint creation of value, future studies should investigate how patients, healthcare practitioners and managers, companies, and regulatory bodies interact to create mutual benefits from the combination of product-service systems. By following this approach, researchers could conduct interviews and observations or apply surveys to get an overview on how different actors collaborate for the co-creation of value, how they perceive and experience the cost-benefits, and what are the drawbacks in collaborations aimed to the prevention of SSIs.
- **DDTs in contaminated and dirty surgeries:** Although empirical evidence suggest that contaminated and dirty surgeries are more likely to result in SSIs, future studies could focus on new technological solutions tailored to mitigate SSI risk factors in these categories. We suggest that research can focus on exploring how DDTs can better help patients to achieve better health conditions in the preoperative stage.
- **Data legitimation and explainability:** Future studies can explore new techniques and methods to promote the acceptance of data generated through DDTs by healthcare practitioners and managers. For example, although the literature on technology acceptance is vast [124], there is still a lack of studies investigating how users accept data generated from DDTs and how to make the data useful for all actors involved in SSI prevention.
- **Data-driven interventions:** Future studies can explore how to design and implement data-driven interventions based on data collected and analysed in the real time. Our review demonstrates that DDTs can be tailored to provide real-time feedback and to promote prompt behavioural change [72]. However, although real-time feedback in healthcare has gained popularity [128], little is known about how to use real-time data to speed up the decision-making process, allowing healthcare practitioners to obtain timely and effective responses and formulate interventions that can be monitored.

## Supplementary Information

Below is the link to the electronic supplementary material.

Supplementary file1 (PDF 94 KB)

Supplementary file2 (PDF 96 KB)

Supplementary file3 (XLSX 19 KB)

## Data Availability

Not applicable.

## References

[1] van Wyk F, Khojandi A, Williams B, MacMillan D, Davis RL, Jacobson DA, Kamaleswaran R (2019). a cost-benefit analysis of automated physiological data acquisition systems using data-driven modeling. J Healthcare Inform Res.

[2] Boursalie O, Samavi R, Doyle TE (2018). Machine learning and mobile health monitoring platforms: a case study on research and implementation challenges. J Healthcare Inform Res.

[3] Lee D (2019). Effects of key value co-creation elements in the healthcare system: focusing on technology applications. Serv Bus.

[4] Kulkov I, Tsvetkova A, Ivanova-Gongne M (2021). Identifying institutional barriers when implementing new technologies in the healthcare industry. Eur J Innov Manag.

[5] Bogers ML, Garud R, Thomas LD, Tuertscher P, Yoo Y (2021) Digital innovation: transforming research and practice*.* Innovation 1–9. 10.1080/14479338.2021.2005465

[6] Núñez-Merino M, Maqueira-Marín JM, Moyano-Fuentes J, Martínez-Jurado PJ (2020). Information and digital technologies of Industry 4.0 and Lean supply chain management: a systematic literature review. Int J Prod Res.

[7] Aceto G, Persico V, Pescapé A (2018). The role of information and communication technologies in healthcare: taxonomies, perspectives, and challenges. J Netw Comput Appl.

[8] Klingenberg CO, Borges MAV, Antunes Jr JAV (2019). Industry 4.0 as a data-driven paradigm: a systematic literature review on technologies. J Manuf Technol Manag.

[9] Trabucchi D, Buganza T (2018). Data-driven innovation: switching the perspective on Big Data. Eur J Innov Manag.

[10] Birkhoff DC, van Dalen ASH, Schijven MP (2021). A review on the current applications of artificial intelligence in the operating room. Surg Innov.

[11] dos Santos R, Silva D, Menezes A, Lukasewicz S, Dalmora C, Carvalho O, Giacomazzi J, Golin N, Pozza R, Vaz T (2021). Automated healthcare-associated infection surveillance using an artificial intelligence algorithm. Infect Prev Pract.

[12] Sawyer RG, Evans HL, Hedrick TL (2019). Technological advances in clinical definition and surveillance methodology for surgical site infection incorporating surgical site imaging and patient-generated health data. Surg Infect.

[13] Hernandez N, Castro L, Medina-Quero J, Favela J, Michan L, Mortenson WB (2021). Scoping review of healthcare literature on mobile, wearable, and textile sensing technology for continuous monitoring. J Healthcare Inform Res.

[14] Scardoni A, Balzarini F, Signorelli C, Cabitza F, Odone A (2020). Artificial intelligence-based tools to control healthcare associated infections: a systematic review of the literature. J Infect Public Health.

[15] Lavallee DC, Lee JR, Semple JL, Lober WB, Evans HL (2019). Engaging patients in co-design of mobile health tools for surgical site infection surveillance: implications for research and implementation. Surg Infect.

[16] Fernandes-Taylor S, Gunter RL, Bennett KM, Awoyinka L, Rahman S, Greenberg CC, Kent KC (2017). Feasibility of implementing a patient-centered postoperative wound monitoring program using smartphone images: a pilot protocol. JMIR Res Protocol.

[17] Mousa AY, Broce M, Davis E, McKee B, Yacoub M (2017). Telehealth electronic monitoring to reduce postdischarge complications and surgical site infections after arterial revascularization with groin incision. J Vasc Surg.

[18] Urbinati A, Manelli L, Frattini F, Bogers ML (2021) The digital transformation of the innovation process: orchestration mechanisms and future research directions*.* Innovation 1–21. 10.1080/14479338.2021.1963736

[19] Byerly S, Maurer LR, Mantero A, Naar L, An G, Kaafarani HM (2021). Machine learning and artificial intelligence for surgical decision making. Surg Infect.

[20] Elfanagely O, Toyoda Y, Othman S, Mellia JA, Basta M, Liu T, Kording K, Ungar L, Fischer JP (2021). Machine learning and surgical outcomes prediction: a systematic review. J Surg Res.

[21] Tranfield D, Denyer D, Smart P (2003). Towards a methodology for developing evidence-informed management knowledge by means of systematic review. Br J Manag.

[22] Owens C, Stoessel K (2008). Surgical site infections: epidemiology, microbiology and prevention. J Hosp Infect.

[23] Cassini A, Plachouras D, Eckmanns T, Abu Sin M, Blank H-P, Ducomble T, Haller S, Harder T, Klingeberg A, Sixtensson M (2016). Burden of six healthcare-associated infections on European population health: estimating incidence-based disability-adjusted life years through a population prevalence-based modelling study. PLoS Med.

[24] Badia J, Casey A, Petrosillo N, Hudson P, Mitchell S, Crosby C (2017). Impact of surgical site infection on healthcare costs and patient outcomes: a systematic review in six European countries. J Hosp Infect.

[25] Cheadle WG (2006). Risk factors for surgical site infection. Surg Infect.

[26] Garner JS (1986). CDC guideline for prevention of surgical wound infections, 1985. Infect Control Hosp Epidemiol.

[27] O’Grady H, Baker E (2011). Prevention of surgical site infections. Surg Infect (Larchmt).

[28] Harrington P (2014). Prevention of surgical site infection. Nurs Stand.

[29] Stockley J, Allen R, Thomlinson D, Constantine C (2001). A district general hospital’s method of post-operative infection surveillance including post-discharge follow-up, developed over a five-year period. J Hosp Infect.

[30] Sibalija J, Barrett D, Subasri M, Bitacola L, Kim RB (2021). Understanding value in a healthcare setting: an application of the business model canvas. Method Innov.

[31] Sweeney JC, Danaher TS, McColl-Kennedy JR (2015). Customer effort in value cocreation activities: improving quality of life and behavioral intentions of health care customers. J Serv Res.

[32] Porter ME (2010). What is value in health care. N Engl J Med.

[33] Porter ME, Teisberg EO (2006). Redefining health care: creating value-based competition on results.

[34] Lakdawalla D, Shafrin J, Lucarelli C, Nicholson S, Khan ZM, Philipson TJ (2015). Quality-adjusted cost of care: a meaningful way to measure growth in innovation cost versus the value of health gains. Health Aff.

[35] Vargo SL, Lusch RF (2004). Evolving to a new dominant logic for marketing. J Mark.

[36] Helkkula A, Kelleher C, Pihlström M (2012). Characterizing value as an experience: implications for service researchers and managers. J Serv Res.

[37] Heinonen K, Strandvik T (2009). Monitoring value-in-use of e-service. J Serv Manag.

[38] Prahalad CK, Ramaswamy V (2004). Co-creation experiences: the next practice in value creation. J Interact Mark.

[39] Secundo G, Shams SR, Nucci F (2021). Digital technologies and collective intelligence for healthcare ecosystem: optimizing Internet of Things adoption for pandemic management. J Bus Res.

[40] Walters D, Jones P (2001). Value and value chains in healthcare: a quality management perspective. TQM Mag.

[41] Spanò R, Massaro M, Iacuzzi S (2021) Blockchain for value creation in the healthcare sector*.* Technovation 102440. 10.1016/j.technovation.2021.102440

[42] Russo Spena T, Cristina M (2020). Practising innovation in the healthcare ecosystem: the agency of third-party actors. J Bus Ind Mark.

[43] Leone D, Schiavone F, Appio FP, Chiao B (2021). How does artificial intelligence enable and enhance value co-creation in industrial markets? An exploratory case study in the healthcare ecosystem. J Bus Res.

[44] Smith JB, Colgate M (2007). Customer value creation: a practical framework. J Market Theory Pract.

[45] Lee C, Coughlin JF (2015). PERSPECTIVE: older adults’ adoption of technology: an integrated approach to identifying determinants and barriers. J Prod Innov Manag.

[46] Magotra I, Sharma J, Sharma SK (2018). Investigating linkage between customer value and technology adoption behaviour: a study of banking sector in India. Eur Res Manag Bus Econ.

[47] Lindman M, Pennanen K, Rothenstein J, Scozzi B, Vincze Z (2016). The value space: how firms facilitate value creation. Bus Process Manag J.

[48] Moher D, Liberati A, Tetzlaff J, Altman DG (2009). PRISMA group: preferred reporting items for systematic reviews and meta-analyses: the PRISMA statement. Ann Intern Med.

[49] Oztemel E, Gursev S (2020). Literature review of Industry 4.0 and related technologies. J Intell Manuf.

[50] de Moura Costa HJ, da Costa CA, da Rosa Righi R, Antunes RS (2020). Fog computing in health: a systematic literature review. Heal Technol.

[51] Schultz A, Goertzen L, Rothney J, Wener P, Enns J, Halas G, Katz A (2018). A scoping approach to systematically review published reviews: adaptations and recommendations. Res Synth Methods.

[52] Braun V, Clarke V (2006). Using thematic analysis in psychology. Qual Res Psychol.

[53] Levac D, Colquhoun H, O'Brien KK (2010). Scoping studies: advancing the methodology. Implement Sci.

[54] Kurmann A, Peter M, Tschan F, Mühlemann K, Candinas D, Beldi G (2011). Adverse effect of noise in the operating theatre on surgical-site infection. J Br Surg.

[55] Nair BG, Newman S-F, Peterson GN, Schwid HA (2011). Automated electronic reminders to improve redosing of antibiotics during surgical cases: comparison of two approaches. Surg Infect.

[56] Campillo-Gimenez B, Garcelon N, Jarno P, Chapplain JM, Cuggia M (2013) Full-text automated detection of surgical site infections secondary to neurosurgery in Rennes, France*.* MEDINFO 572–575. 10.3233/978-1-61499-289-9-572 23920620

[57] Michelson JD, Pariseau JS, Paganelli WC (2014). Assessing surgical site infection risk factors using electronic medical records and text mining. Am J Infect Control.

[58] Zhao T, Zhang X, Zeng L, Xia S, Hinton AO, Li X (2014). Applications for radio-frequency identification technology in the perioperative setting. AORN J.

[59] Du M, Xing Y, Suo J, Liu B, Jia N, Huo R, Chen C, Liu Y (2014). Real-time automatic hospital-wide surveillance of nosocomial infections and outbreaks in a large Chinese tertiary hospital. BMC Med Inform Decis Mak.

[60] Simons FE, Aij KH, Widdershoven GA, Visse M (2014) Patient safety in the operating theatre: how A3 thinking can help reduce door movement*.* Int J Qual Health Care 366–371. http://www.jstor.org/stable/45128004 10.1093/intqhc/mzu033 24699198

[61] van Esbroeck A, Rubinfeld I, Hall B, Syed Z (2014). Quantifying surgical complexity with machine learning: looking beyond patient factors to improve surgical models. Surgery.

[62] Hu Z, Simon GJ, Arsoniadis EG, Wang Y, Kwaan MR, Melton GB (2015). Automated detection of postoperative surgical site infections using supervised methods with electronic health record data. Stud Health Technol Inform.

[63] Esser J, Shrinski K, Cady R, Belew J (2016). Reducing OR traffic using education, policy development, and communication technology. AORN J.

[64] Hwang H (2016) Electronic wound monitoring after ambulatory breast cancer surgery: improving patient care and satisfaction using a smart phone app*.* BCMJ 58(8):448–453. https://bcmj.org/articles/electronic-wound-monitoring-after-ambulatory-breast-cancer-surgery-improving-patient-care

[65] Gunter R, Fernandes-Taylor S, Mahnke A, Awoyinka L, Schroeder C, Wiseman J, Sullivan S, Bennett K, Greenberg C, Kent KC (2016). Evaluating patient usability of an image-based mobile health platform for postoperative wound monitoring. JMIR mHealth and uHealth.

[66] Sanger PC, van Ramshorst GH, Mercan E, Huang S, Hartzler AL, Armstrong CA, Lordon RJ, Lober WB, Evans HL (2016). A prognostic model of surgical site infection using daily clinical wound assessment. J Am Coll Surg.

[67] Sanger PC, Hartzler A, Lordon RJ, Armstrong CA, Lober WB, Evans HL, Pratt W (2016). A patient-centered system in a provider-centered world: challenges of incorporating post-discharge wound data into practice. J Am Med Inform Assoc.

[68] Wiseman JT, Fernandes-Taylor S, Gunter R, Barnes ML, Saunders RS, Rathouz PJ, Yamanouchi D, Kent KC (2016). Inter-rater agreement and checklist validation for postoperative wound assessment using smartphone images in vascular surgery. J Vasc Surg Venous Lymphat Disord.

[69] Ke C, Jin Y, Evans H, Lober B, Qian X, Liu J, Huang S (2017). Prognostics of surgical site infections using dynamic health data. J Biomed Inform.

[70] Sohn S, Larson DW, Habermann EB, Naessens JM, Alabbad JY, Liu H (2017). Detection of clinically important colorectal surgical site infection using Bayesian network. J Surg Res.

[71] Sanger PC, Simianu VV, Gaskill CE, Armstrong CA, Hartzler AL, Lordon RJ, Lober WB, Evans HL (2017). Diagnosing surgical site infection using wound photography: a scenario-based study. J Am Coll Surg.

[72] Eskildsen SM, Moskal PT, Laux J, Gaizo DJD (2017). The effect of a door alarm on operating room traffic during total joint arthroplasty. Orthopedics.

[73] Weller GB, Lovely J, Larson DW, Earnshaw BA, Huebner M (2018). Leveraging electronic health records for predictive modeling of post-surgical complications. Stat Methods Med Res.

[74] Falen T, Noblin AM, Russell OL, Santiago N (2018). Using the electronic health record data in real time and predictive analytics to prevent hospital-acquired postoperative/surgical site infections. Health News.

[75] Gunter RL, Fernandes-Taylor S, Rahman S, Awoyinka L, Bennett KM, Weber SM, Greenberg CC, Kent KC (2018). Feasibility of an image-based mobile health protocol for postoperative wound monitoring. J Am Coll Surg.

[76] Armellino D, Dowling O, Newman SB, Schwarz RB, Jacobs M, Cifu-Tursellino K, Di Capua JF (2018). Remote video auditing to verify OR cleaning: a quality improvement project. AORN J.

[77] Ribed A, Monje B, García-González X, Sanchez-Somolinos M, Sanz-Ruiz P, Rodríguez-González CG, Sanjurjo-Saez M (2020). Improving surgical antibiotic prophylaxis adherence and reducing hospital readmissions: a bundle of interventions including health information technologies. Eur J Hosp Pharm.

[78] Canty M, St George EJ (2018). Development of a surgical site infection surveillance programme in a Scottish neurosurgical unit. Int J Health Gov.

[79] Broman KK, Gaskill CE, Faqih A, Feng M, Phillips SE, Lober WB, Pierce RA, Holzman MD, Evans HL, Poulose BK (2019). Evaluation of wound photography for remote postoperative assessment of surgical site infections. JAMA Surg.

[80] Lu K, Chermside-Scabbo CJ, Marino NE, Concepcion A, Yugawa C, Aladegbami B, Paar T, St John TA, Ross W, Clohisy JC (2018). Accessible communication tools for surgical site infection monitoring and prevention in joint reconstruction: feasibility study. JMIR Perioper Med.

[81] Rosner BI, Gottlieb M, Anderson WN (2018). Accuracy of internet-based patient self-report of postdischarge health care utilization and complications following orthopedic procedures: observational cohort study. J Med Internet Res.

[82] Weiser MC, Shemesh S, Chen DD, Bronson MJ, Moucha CS (2018). The effect of door opening on positive pressure and airflow in operating rooms. JAAOS-J Am Acad Orthop Surg.

[83] Bartz-Kurycki MA, Green C, Anderson KT, Alder AC, Bucher BT, Cina RA, Jamshidi R, Russell RT, Williams RF, Tsao K (2018). Enhanced neonatal surgical site infection prediction model utilizing statistically and clinically significant variables in combination with a machine learning algorithm. Am J Surg.

[84] Mousa AY, Broce M, Monnett S, Davis E, McKee B, Lucas BD (2019). Results of telehealth electronic monitoring for post discharge complications and surgical site infections following arterial revascularization with groin incision. Ann Vasc Surg.

[85] Childs C, Wright N, Willmott J, Davies M, Kilner K, Ousey K, Soltani H, Madhuvrata P, Stephenson J (2019). The surgical wound in infrared: thermographic profiles and early stage test-accuracy to predict surgical site infection in obese women during the first 30 days after caesarean section. Antimicrob Resist Infect Control.

[86] Hsu J-T, Chen Y-W, Ho T-W, Tai H-C, Wu J-M, Sun H-Y, Hung C-S, Zeng Y-C, Kuo S-Y, Lai F (2019). Chronic wound assessment and infection detection method. BMC Med Inform Decis Mak.

[87] Birgand G, Azevedo C, Rukly S, Pissard-Gibollet R, Toupet G, Timsit J-F, Lucet J-C, AS Group (2019). Motion-capture system to assess intraoperative staff movements and door openings: impact on surrogates of the infectious risk in surgery. Infect Control Hosp Epidemiol.

[88] Zhang J, Dushaj K, Rasquinha VJ, Scuderi GR, Hepinstall MS (2019). Monitoring surgical incision sites in orthopedic patients using an online physician-patient messaging platform. J Arthroplasty.

[89] Shen F, Larson DW, Naessens JM, Habermann EB, Liu H, Sohn S (2019). Detection of surgical site infection utilizing automated feature generation in clinical notes. J Healthcare Inform Res.

[90] Gowd AK, Agarwalla A, Amin NH, Romeo AA, Nicholson GP, Verma NN, Liu JN (2019). Construct validation of machine learning in the prediction of short-term postoperative complications following total shoulder arthroplasty. J Shoulder Elbow Surg.

[91] Haskins IN, Olson MA, Stewart TG, Rosen MJ, Poulose BK (2019). Development and validation of the ventral hernia repair outcomes reporting app for clinician and patient engagement (ORACLE). J Am Coll Surg.

[92] Tunthanathip T, Sae-Heng S, Oearsakul T, Sakarunchai I, Kaewborisutsakul A, Taweesomboonyat C (2019). Machine learning applications for the prediction of surgical site infection in neurological operations. Neurosurg Focus.

[93] da Silva DA, Ten Caten CS, Dos Santos RP, Fogliatto FS, Hsuan J (2019). Predicting the occurrence of surgical site infections using text mining and machine learning. PloS One.

[94] Azevedo-Coste C, Pissard-Gibollet R, Toupet G, Fleury É, Lucet J-C, Birgand G (2019). Tracking clinical staff behaviors in an operating room. Sensors.

[95] Azimi K, Honaker MD, Chalil Madathil S, Khasawneh MT (2020). Post-operative infection prediction and risk factor analysis in colorectal surgery using data mining techniques: a pilot study. Surg Infect.

[96] Bucher BT, Shi J, Ferraro JP, Skarda DE, Samore MH, Hurdle JF, Gundlapalli AV, Chapman WW, Finlayson SR (2020). Portable automated surveillance of surgical site infections using natural language processing: development and validation. Ann Surg.

[97] Chang B, Sun Z, Peiris P, Huang ES, Benrashid E, Dillavou ED (2020). Deep learning-based risk model for best management of closed groin incisions after vascular surgery. J Surg Res.

[98] Chen W, Lu Z, You L, Zhou L, Xu J, Chen K (2020). Artificial intelligence–based multimodal risk assessment model for surgical site infection (AMRAMS): development and validation study. JMIR Med Inform.

[99] CiofiDegliAtti ML, Pecoraro F, Piga S, Luzi D, Raponi M (2020). Developing a surgical site infection surveillance system based on hospital unstructured clinical notes and text mining. Surg Infect.

[100] Hopkins BS, Mazmudar A, Driscoll C, Svet M, Goergen J, Kelsten M, Shlobin NA, Kesavabhotla K, Smith ZA, Dahdaleh NS (2020). Using artificial intelligence (AI) to predict postoperative surgical site infection: a retrospective cohort of 4046 posterior spinal fusions. Clin Neurol Neurosurg.

[101] Karhade AV, Bongers ME, Groot OQ, Cha TD, Doorly TP, Fogel HA, Hershman SH, Tobert DG, Schoenfeld AJ, Kang JD (2020). Can natural language processing provide accurate, automated reporting of wound infection requiring reoperation after lumbar discectomy?. Spine J.

[102] Park HJ, Jung DY, Ji W, Choi C-M (2020). Detection of bacteremia in surgical in-patients using recurrent neural network based on time series records: development and validation study. J Med Internet Res.

[103] Park HJ, min Kim S, Kim HR, Ji W, Choi C-M (2020). The value of preoperative spirometry testing for predicting postoperative risk in upper abdominal and thoracic surgery assessed using big-data analysis. J Thorac Dis.

[104] Schlund D, Poirier J, Bhama AR, Hayden D, Saclarides T, Orkin B, Favuzza J (2020). Value of an interactive phone application in an established enhanced recovery program. Int J Colorectal Dis.

[105] van Niekerk J, Vos M, Stein A, Braakman-Jansen L, Voor in ‘t holt A, van Gemert-Pijnen J (2020). Risk factors for surgical site infections using a data-driven approach. PloS One.

[106] Formeister EJ, Baum R, Knott PD, Seth R, Ha P, Ryan W, El-Sayed I, George J, Larson A, Plonowska K (2020). Machine learning for predicting complications in head and neck microvascular free tissue transfer. Laryngoscope.

[107] Elhage SA, Deerenberg EB, Ayuso SA, Murphy KJ, Shao JM, Kercher KW, Smart NJ, Fischer JP, Augenstein VA, Colavita PD (2021). Development and validation of image-based deep learning models to predict surgical complexity and complications in abdominal wall reconstruction. JAMA Surg.

[108] McLean KA, Mountain KE, Shaw CA, Drake TM, Pius R, Knight SR, Fairfield CJ, Sgrò A, Bouamrane M, Cambridge WA (2021). Remote diagnosis of surgical-site infection using a mobile digital intervention: a randomised controlled trial in emergency surgery patients. NPJ Digit Med.

[109] Petrosyan Y, Thavorn K, Smith G, Maclure M, Preston R, van Walravan C, Forster AJ (2021). Predicting postoperative surgical site infection with administrative data: a random forests algorithm. BMC Med Res Methodol.

[110] Shahroudi P, Aarabi A (2021). Quality improvement through lean A3 method for foot traffic in operating room. Perioper Care Oper Room Manag.

[111] Wang Y, Zhang D, Wei S (2021) Effect of nursing intervention in the operating room based on simple virtual reality augmented technology on preventing gastrointestinal surgical incision infection*.* J Healthcare Eng 2021. 10.1155/2021/9981821 10.1155/2021/9981821 PMC8110406 34007434

[112] Zhu Y, Simon GJ, Wick EC, Abe-Jones Y, Najafi N, Sheka A, Tourani R, Skube SJ, Hu Z, Melton GB (2021). Applying machine learning across sites: external validation of a surgical site infection detection algorithm. J Am Coll Surg.

[113] Soenksen LR, Yazdi Y (2017). Stage-gate process for life sciences and medical innovation investment. Technovation.

[114] Cohen AB, Dorsey E, Mathews SC, Bates DW, Safavi K (2020). A digital health industry cohort across the health continuum. NPJ Digital Medicine.

[115] Cook E, Marchaim D, Kaye KS (2011). Building a successful infection prevention program: key components, processes, and economics. Infect Dis Clin.

[116] Garcell HG, Arias AV, Sandoval CAP, García EG, Gamboa MEV, Sado AB, Serrano RNA (2017). Incidence and etiology of surgical site infections in appendectomies: a 3-year prospective study. Oman Med J.

[117] Carvalho RLRd, Campos CC, Franco LMdC, Rocha ADM, Ercole FF (2017) Incidence and risk factors for surgical site infection in general surgeries 1. Revista Latino-Americana de Enfermagem 25. 10.1590/1518-8345.1502.2848 10.1590/1518-8345.1502.2848 PMC5738868 29211190

[118] Mankins JC (2009). Technology readiness assessments: a retrospective. Acta Astronaut.

[119] Khazaei H, McGregor C, Eklund JM, El-Khatib K (2015). Real-time and retrospective health-analytics-as-a-service: a novel framework. JMIR Med Inform.

[120] Kirby JP, Mazuski JE (2009). Prevention of surgical site infection. Surg Clin North Am.

[121] Dellinger EP, Villaflor-Camagong D, Whimbey E (2021). Gradually increasing surgical site infection prevention bundle with monitoring of potentially preventable infections resulting in decreasing overall surgical site infection rate. Surg Infect.

[122] Gbegnon A, Monestina J, Cromwell J (2014). Machine learning algorithm for accurate, automated, real-time prediction of surgical site infections using EHR data. J Surg Res.

[123] Greenhalgh T, Wherton J, Papoutsi C, Lynch J, Hughes G, Hinder S, Fahy N, Procter R, Shaw S (2017). Beyond adoption: a new framework for theorizing and evaluating nonadoption, abandonment, and challenges to the scale-up, spread, and sustainability of health and care technologies. J Med Internet Res.

[124] King WR, He J (2006). A meta-analysis of the technology acceptance model. Inform Manag.

[125] Kumar M, Singh JB, Chandwani R, Gupta A (2020). “Context” in healthcare information technology resistance: a systematic review of extant literature and agenda for future research. Int J Inform Manag.

[126] Yip MH, Phaal R, Probert DR (2015). Characterising product-service systems in the healthcare industry. Technol Soc.

[127] Peng Y, Wu T, Chen Z, Deng Z (2022). Value cocreation in health care: systematic review. J Med Internet Res.

[128] Maktoubian J, Ansari K (2019). An IoT architecture for preventive maintenance of medical devices in healthcare organizations. Heal Technol.

