# Supplementary material for: Data-Driven Technologies as Enablers for Value Creation in the Prevention of Surgical Site Infections: a Systematic Review
Source: J Healthc Inform Res. 2023 Feb 27;7(1):1–41. doi: 10.1007/s41666-023-00129-2 (PMC9995622; doi:10.1007/s41666-023-00129-2)
Supplement: Supplementary file 1 — Supplementary file1 (PDF 94 KB) [file 41666_2023_129_MOESM1_ESM.pdf]

Electronic supplementary material 1: Search parameters

Main concept	Alternative concept / search terms	Search within
Surgical site infection	Surgical site infection<br>Surgical wound infection<br>Postoperative infection	Article title, Abstract and Key words
Data-driven technology	Information and communication technology<br>Data-driven<br>Digital<br>Internet of Things (or IoT)<br>Artificial intelligence (or AI)<br>Machine learning (or ML)<br>Cloud computing<br>Big data<br>Deep learning<br>Data mining<br>Sensors<br>Smart<br>Virtual Reality (or VR)<br>Radio frequency identification (or RFID)<br>Natural language processing (or NLP)<br>Augmented reality<br>Robot<br>Mobile<br>Video<br>Blockchain	Article title, Abstract and Key words

Elaborated by the authors of the study.
