# Supplementary material for: Data-Driven Technologies as Enablers for Value Creation in the Prevention of Surgical Site Infections: a Systematic Review
Source: J Healthc Inform Res. 2023 Feb 27;7(1):1–41. doi: 10.1007/s41666-023-00129-2 (PMC9995622; doi:10.1007/s41666-023-00129-2)
Supplement: Supplementary file 2 — Supplementary file2 (PDF 96 KB) [file 41666_2023_129_MOESM2_ESM.pdf]

Electronic supplementary material 2: Illustration of database search.

Database	Alternative concept / search terms	Items found*
Scopus	TITLE-ABS-KEY (("surgical site infection") OR ("surgical wound infection") OR ("postoperative infection")) AND TITLE-ABS-KEY ((information PRE/2 technology) OR ("data-driven") OR (digit*) OR ("Internet of Things") OR ("artificial intelligence") OR ("machine learning") OR ("cloud computing") OR ("natural language process") OR ("big data") OR ("deep learning") OR ("data mining") OR (sensor) OR ("virtual reality") OR ("augmented reality") OR ("radio frequency identification") OR (robot*) OR (mobile) OR (video) OR ("blockchain") OR (smart))	1061
We of Science	AB=((surgical NEAR/2 infection) OR (postoperative NEAR/2 infection)) AND AB=((information NEAR/2 technology) OR ("data-driven") OR (digit*) OR ("Internet of Things") OR ("artificial intelligence") OR ("machine learning") OR ("cloud computing") OR ("natural language process") OR ("big data") OR ("deep learning") OR ("data mining") OR (sensor) OR ("virtual reality") OR ("augmented reality") OR ("radio frequency identification") OR (robot*) OR (mobile) OR (video) OR ("blockchain") OR (smart))	451
MEDLINE (EBSCO)	AB(("surgical site infection") OR ("surgical wound infection") OR ("postoperative infection")) AND (AB (("information and communication technology") OR ("data-driven") OR (digit*) OR ("Internet of Things") OR ("artificial intelligence") OR ("machine learning") OR ("cloud computing") OR ("natural language process") OR ("big data") OR ("deep learning") OR ("data mining") OR (sensor) OR ("virtual reality") OR ("augmented reality") OR ("radio frequency identification") OR (robot*) OR (mobile) OR (video) OR ("blockchain") OR (smart))	249
ProQuest	(("surgical site infection") OR ("surgical wound infection") OR ("postoperative infection")) AND (("data-driven") OR (digital) OR ("Internet of Things") OR ("artificial intelligence") OR ("machine learning") OR ("cloud computing") OR ("natural language process") OR ("big data") OR ("deep learning") OR ("data mining") OR (sensor) OR ("virtual reality") OR ("augmented reality") OR ("radio frequency identification") OR (robot) OR (mobile) OR (video) OR ("blockchain") OR (smart))	125
PubMed	(("surgical site infection") OR ("surgical wound infection") OR ("postoperative infection") [Title/Abstract]) AND (("information and communication technology") OR ("data-driven") OR (digit*) OR ("Internet of Things") OR ("artificial intelligence") OR ("machine learning") OR ("cloud computing") OR ("natural language process") OR ("big data") OR ("deep learning") OR ("data mining") OR (sensor) OR ("virtual reality") OR ("augmented reality") OR ("radio frequency identification") OR (robot*) OR (mobile) OR (video) OR ("blockchain") OR (smart) [Title/Abstract])	536
ABI/Inform Global	ab("surgical site infection") OR ("surgical wound infection") OR ("postoperative infection")) AND (ab (("information and communication technology") OR ("data-driven") OR (digit*) OR ("Internet of Things") OR ("artificial intelligence") OR ("machine learning") OR ("cloud computing") OR ("natural language process") OR ("big data") OR ("deep learning") OR ("data mining") OR (sensor) OR ("virtual reality") OR ("augmented reality") OR ("radio frequency identification") OR (robot*) OR (mobile) OR (video) OR ("blockchain") OR (smart))	81
Cochrane	((“surgical site infection”):ti,ab,kw OR (“surgical wound infection”):ti,ab,kw OR (“postoperative infection”):ti,ab,kw) AND (("information and communication technology"):ti,ab,kw OR ("big data"):ti,ab,kw OR ("machine learning"):ti,ab,kw OR ("artificial intelligence"):ti,ab,kw OR ("internet of things"):ti,ab,kw OR ("deep learning"):ti,ab,kw OR ("blockchain"):ti,ab,kw OR ("cloud computing"):ti,ab,kw OR ("data mining"):ti,ab,kw OR ("smart sensor"):ti,ab,kw OR ("wireless"):ti,ab,kw OR ("natural language process"):ti,ab,kw OR (digital):ti,ab,kw OR (ICT):ti,ab,kw OR (data driven):ti,ab,kw OR (video):ti,ab,kw OR (mobile):ti,ab,kw OR (digit*):ti,ab,kw)	94
**Total**		**2597**

Elaborated by the authors of the study.
